# GPR171 expression enhances proliferation and metastasis of lung cancer cells

**DOI:** 10.18632/oncotarget.6856

**Published:** 2016-01-09

**Authors:** So Hee Dho, Kwang-Pyo Lee, Dongjun Jeong, Chang-Jin Kim, Kyung-Sook Chung, Ji Young Kim, Bum-Chan Park, Sung Sup Park, Seon-Young Kim, Ki-Sun Kwon

**Affiliations:** ^1^ Aging Research Institute, Korea Research Institute of Bioscience and Biotechnology, Daejeon 305-806, Republic of Korea; ^2^ Radioisotope Research Division, Department of Research Reactor Utilization, Korea Atomic Energy Research Institute, Daejeon 305-353, Republic of Korea; ^3^ Department of Pathology, College of Medicine Soonchunhyang University, Chonan 330-090, Republic of Korea; ^4^ Genome Research Center, Korea Research Institute of Bioscience and Biotechnology, Daejeon 305-806, Republic of Korea; ^5^ Department of Functional Genomics, Korea University of Science and Technology (UST), Daejeon 305-333, Republic of Korea

**Keywords:** GPCR, GPR171, EGFR, lung cancer

## Abstract

G protein-coupled receptors (GPCRs) are among the most significant therapeutic targets and some of them promote the growth and metastasis of cancer. Here, we show that an increase in the levels of GPR171 is crucial for lung cancer tumor progression *in vitro* and *in vivo*. Immunostaining of clinical samples indicated that GPR171 was overexpressed in 46.8% of lung carcinoma tissues. Depletion of GPR171 with an anti-GPR171 antibody decreased proliferation of lung carcinoma cells and attenuated tumor progression in a mouse xenograft model. Knockdown of GPR171 also inhibited migration and invasion of the lung cancer cell lines. Notably, inhibition of GPR171 synergistically enhanced the tumoricidal activity of an epidermal growth factor receptor (EGFR) inhibitor in lung cancer cells. These results indicate that GPR171 blockade is a promising antineoplastic strategy and provide a preclinical rationale for combined inhibition of GPR171 and EGFR.

## INTRODUCTION

Lung and bronchus cancers account for the greatest proportion of cancer deaths worldwide [1, 2]. Moreover, improvement in survival over the past several decades is lower for lung cancer than all other cancers [2]. Therefore, considerable effort has been focused on the identification of effective drug targets for lung cancer. There are two clinical types of lung cancer: non-small cell lung carcinoma (NSCLC), which accounts for 85% of all cases, and small cell lung carcinoma (SCLC) [1, 3]. NSCLC can be further divided into three main subtypes: squamous cell carcinoma (30% of all lung cancers); adenocarcinoma (40% of all lung cancers), including bronchioloalveolar carcinoma; and large cell carcinoma (5–15% of all lung cancers) [4, 5]. EGFR (epidermal growth factor receptor), ALK (anaplastic lymphoma receptor tyrosine kinase), RET (ret proto-oncogene receptor tyrosine kinase), and BRAF (B-Raf proto-oncogene, serine/threonine kinase) are well-identified oncogenic driver mutations in lung cancer, and are targets for therapeutic application.

In contrast to the case for SCLC, there have been therapeutic breakthroughs in NSCLC, one of which is the EGFR inhibitor [3]. Clinical trials have revealed variability in the response to gefitinib (‘Iressa’, ZD1839), an EGFR inhibitor, with higher responses seen in Asian-derived populations (27.5%) compared with European-derived populations (10.4%) [6]. The disappointing response to EGFR inhibitors among Caucasians is likely related to mutations in the EGFR [6]. Therefore, different therapies targeting factors other than EGFR could compensate for the efficacy of EGFR inhibitors [7].

G protein-coupled receptors (GPCRs), which share a characteristic seven-transmembrane-domain topology, can be divided into five families based on their sequence homology [8, 9]. There are approximately 1,000 members of the GPCR superfamily, many of which serve functions that remain to be identified [10]. Some GPCRs have been implicated as oncogenes, and many are reported to contribute to the growth and metastasis of lung cancer [11]. For example, in the case of NSCLC, prostanoid (EP) receptors [12–15] and the C-X-C motif chemokine receptor CXCR2 [16, 17] are involved in growth, metastasis and angiogenesis, whereas CXCR4 [18, 19] affects migration and metastasis. In the case of SCLC, GRPR (gastrin-releasing peptide receptor) [20, 21] and NMBR (neuromedin B receptor) [22] contribute to growth; cholecystokinin-1 (CCK1) and -2 (CCK2) [23] mediate growth and survival; and CXCR4 [24] induces growth and metastasis.

Through a bioinformatics approach, we identified GPR171, a member of the class A rhodopsin superfamily, specifically the P2Y12 family [25], as a potential tumor-promoting gene that enhanced proliferation and metastasis of lung cancer. The biological role of GPR171 has recently started to come into focus, and found to involve feeding and metabolism in mice [26] and myeloid differentiation [27]. Our data further suggest that GPR171 is a promising target for the development of antineoplastic drugs.

## RESULTS

### GPR171 is a novel gene overexpressed in lung cancer

**Figure 1:**
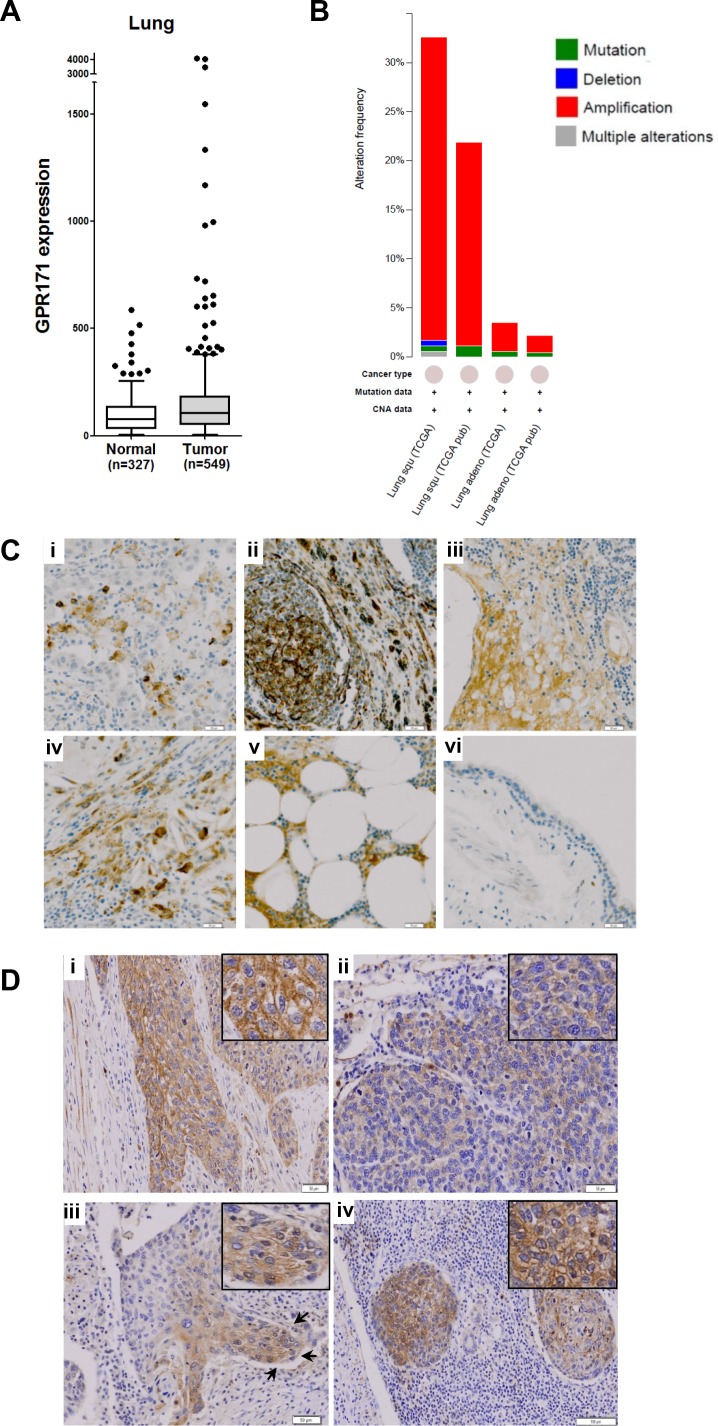
GPR171 expression is induced in lung cancer tissues **A.** GPR171 mRNA expression in normal lung and lung cancer tissues. Expression was assessed using the GENT database [38]. Boxes indicate the 75^th^ percentile, median, and 25^th^ percentile. Dots indicate outliers. **B.** Alteration frequency of *GPR171* in lung squamous cell carcinoma (Lung squ) and adenocarcinoma (Lung adeno). **C.** Immunohistochemical analysis of GPR171 in lung cancer tissues.(i) Squamous cell carcinoma of the lung; (ii) adenocarcinoma of the lung; (iii) small-cell lung carcinoma; (iv) large-cell lung carcinoma; (v) lymph nodal metastatic carcinoma from adenocarcinoma of the lung; (vi) normal bronchial epithelium. Scale bars = 50 μm. **D.** Immunohistochemical analysis of GPR171 in (i) well-differentiated squamous cell carcinoma. Scale bars = 50 μm; (ii) poorly-differentiated squamous cell carcinoma. Scale bars = 50 μm; (iii) the invading front of squamous cell carcinoma. Arrows indicate invading tumor fronts in squamous cell carcinoma. Scale bars = 100 μm; (iv) lymph nodal metastatic tumor cells. Scale bars = 100 μm. Insets show magnified images to present GPR171 is expressed in the cytoplasm and cytoplasmic membrane.

Because critical oncogenes are markedly overexpressed in a subset of cancer cases rather than across all cancer cases, a cancer outlier profile analysis is required to identify pro-tumorigenic targets [28]. For example, ERBB2 (HER2), a well-known proto-oncogene, is overexpressed owing to gene amplification in 25–30% of invasive ductal breast carcinomas [29]. To identify pro-tumorigenic targets, we applied a cancer outlier profile analysis to the GENT microarray database containing 16,400 samples from the Affymetrix U133A platform. Among cancer outlier candidates, GPCRs were our focus because they have been implicated in tumor growth and are aberrantly overexpressed in tumor cells [11]. Among the 703 GPCR family members in our database, we selected an initial pool of candidate genes based on three criteria: (1) the target gene is overexpressed in tumor tissues, compared to the normal counterparts from the GENT database search (medical-genome.kribb.re.kr/GENT/), (2) the target gene is overexpressed in a subset of cancer cell lines from the same tissue, and (3) the target gene has not been extensively studied, as ascertained from the literature search. Based on the above criteria, three genes (GPR171, GABBR1, and GPR88) were selected for further analyses. GPR171 was overexpressed in subsets of breast, liver, lung, and ovarian tumors (Figure 1A and Supplementary Figure 1A), along with a subset of cancer cell lines from the lung (Supplementary Figure 1B). To our knowledge, GPR171 is a novel target in cancer. To further confirm that GPR171 is indeed overexpressed in lung cancers, we searched for *GPR171* gene alterations in lung tumors using the cBioPortal for cancer genomics [30, 31]. Interestingly, we found increased gene copy numbers rather than mutations of *GPR171* in lung squamous cell carcinoma and adenocarcinoma (Figure 1B). These results suggest a role for GPR171 as a putative tumor-promoting gene.

Since microarray data do not always reflect the pattern of protein expression in tissues, we performed an immunohistochemical assessment of lung cancer tissues. The specificity of the antibody was confirmed via flow cytometry analysis using GPR171-negative MDA-MB-231 cells with or without ectopic expression of GPR171 (Supplementary Figure 2). The immunohistochemisty analysis showed that GPR171 was expressed in various lung cancer tissues, but was rarely detected in normal bronchial epithelium (Table 1). Of 47 lung cancer tissues, 22 (46.8%) were positive for GPR171; of 35 NSCLC specimens, 16 (45.7%) stained positively for GPR171 expression (Table 1). Among NSCLC subtypes, squamous cell carcinoma, bronchioloalveolar carcinoma, adenosquamous carcinoma, and large-cell carcinoma exhibited a relatively high frequency of positive immunostaining for GPR171 (Supplementary Table 1), whereas mucoepidermoid carcinoma did not. We were able to determine that GPR171 expression was also elevated in SCLC, even given the limited sample size (Supplementary Table 2). Representative GPR171-positive cases, including squamous cell carcinoma, adenocarcinoma, small-cell lung carcinoma, large-cell lung carcinoma and lymph nodal metastatic carcinoma from adenocarcinomas of the lung, are shown in Figure 1C. Immunohistochemistry showed that Gpr171 was expressed in the cytoplasmic membrane and cytoplasm of NSCLC (Figure 1D, insets). The expression was higher in well-differentiated than in poorly-differentiated squamous cell carcinoma (Figure 1D, i and ii). Moreover, Gpr171 was highly expressed in the invading front of squamous cell carcinoma (Figure 1D, iii) as well as in cells metastatic to the lymph node (Figure 1D, iv). GPR171 was detected in only two cases out of nine normal bronchial epithelium (Table 1 and Figure 1C, vi). Although not statistically significant (by Fisher's exact test), all the three cancer types (NSCLC, SCLC, and metastasis) showed higher positive staining than normal samples. This preferential expression in cancer tissues, which is central to the issue of clinical relevance, suggests that GPR171 is a pro-tumorigenic target in lung cancer.

**Table 1:** GPR171 is upregulated in a subset of lung cancer tissues

Clinical types of lung cancer	Number of stained samples/examined samples			Total positive tissues
	Strong	Moderate	Negative	
**Cancer**	8/47	14/47	25/47	22/47 (46.8%)
NSCLC	4/35	12/35	19/35	16/35 (45.7%)
SCLC	1/3	1/3	1/3	2/3
Metastasis	3/9	1/9	5/9	4/9
**Normal**	0/9	2/9	7/9	2/9

Immunohistochemical quantification of GPR171 in lung cancer and normal tissues. Strong, moderate, and negative indicate >50%, 10-50%, and <10% positive cells, respectively.

### GPR171 triggers proliferation of lung cancer cells *in vitro* and *in vivo*

**Figure 2:**
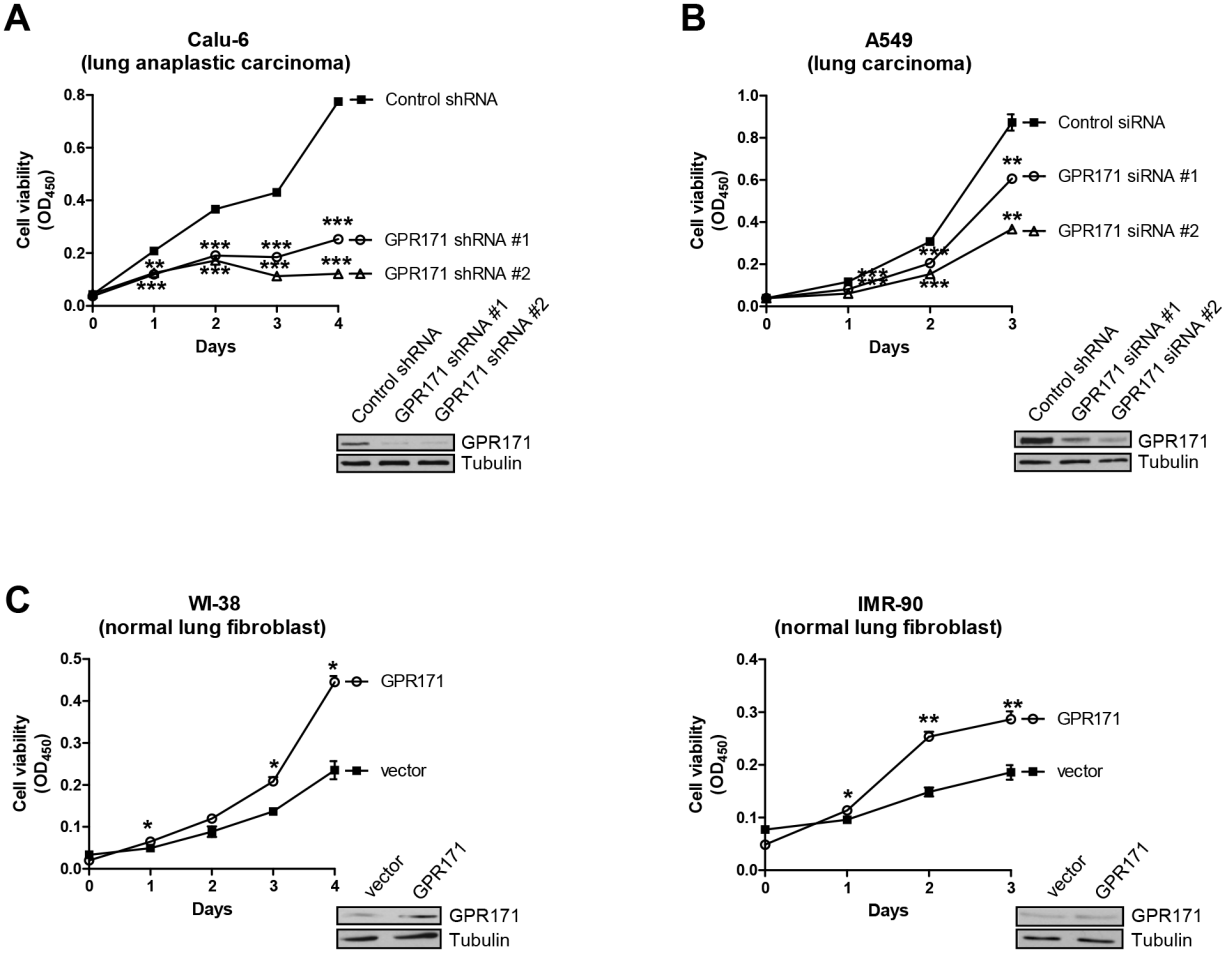
GPR171 promotes proliferation of lung cancer cells **A.** Cell viability assays of Calu-6 lung cancer cells expressing control or two different GPR171 shRNAs (n = 3; **P < 0.01, ***P < 0.001 vs. control shRNA, Student's t test). **B.** Cell viability assays of A549 lung cancer cells expressing control or two different GPR171 siRNAs. Results are presented as means ± SEM (error bars) (n = 3; **P < 0.01, ***P < 0.001 vs. control siRNA, Student's t test). **C.** Cell viability assays of WI-38, and IMR-90 cells expressing control vector or a GPR171 expression plasmid. Results are presented as means ± SEM (error bars) (n = 3; *P < 0.05, **P < 0.01 vs. vector control, Student's t test). Error bars on the values were smaller than the symbols. Knockdown or ectopic expression of GPR171 was confirmed by immunoblotting (insets). Tubulin was used as a loading control.

Having demonstrated that GPR171 is preferentially expressed in various lung cancer tissues, we next sought to confirm a functional role of GPR171 in lung cancer using an RNA interference approach. To determine if GPR171 has a role in cellular proliferation, we examined the growth rates of an NSCLC cancer cell line, Calu-6 (lung anaplastic carcinoma), expressing one of two different small hairpin RNAs (shRNAs) against GPR171 or control shRNA. GPR171 silencing with shRNA #1 or #2 significantly suppressed cell proliferation, reducing the growth of Calu-6 cells to 32.7% (p = 8.4×10^−4^) and 16.7% (p = 2.2×10^−4^) of that in control shRNA cells, respectively, 4 days after infection (Figure 2A). Next, we investigated whether GPR171 siRNAs targeting other sites in the *GPR171* sequence inhibited A549 (lung carcinoma) cell proliferation. Consistent with Calu-6 cell results, A549 cells expressing GPR171 siRNA #1 or #2 grew ∼30% (p = 6.3×10^−3^) and ∼65% (p = 3.3×10^−3^) less than cells expressing control siRNA 3 days after transfection (Figure 2B).

To further confirm the role of GPR171 in cellular proliferation, we examined the proliferative effect of ectopic expression of GPR171 in normal lung cells. As expected, overexpression of GPR171 in WI-38 and IMR-90 normal lung fibroblast cell lines enhanced cell proliferation, increasing growth by 47.1% (p = 0.014) and 35.2% (p = 6.4×10^−3^), respectively, compared with that of cells expressing control siRNA (Figure 2C).

**Figure 3:**
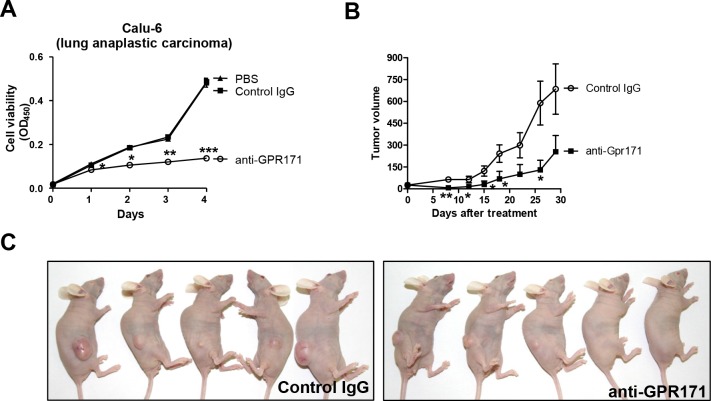
GPR171 triggers proliferation of lung cancer cells in vivo **A.** Cell viability assays of Calu-6 cells treated with anti-GPR171 antibody (ab60843; Abcam), IgG control antibody, or vehicle (PBS) control. Results are presented as means ± SEM (error bars) (n = 3; *P < 0.05, **P < 0.01, ***P < 0.001 vs. control IgG, Student's t test). **B.** Effects of anti-GPR171 antibody on the growth of Calu-6 xenograft tumors. Data are presented as mean tumor volume ± SEM (error bars) (n = 5; *P < 0.05, **P < 0.01 vs. control IgG, Student's t test). **C.** BALB/c nude mice bearing Calu-6 xenograft tumors treated with control or anti-GPR171 antibodies.

To validate GPR171 as an anticancer target, we challenged GPR171-positive cancer cells with GPR171-specific antibodies. The use of antibodies is an established cancer therapy approach, with antibodies targeting EGFR, ERBB2, and VEGF (vascular endothelial growth factor) demonstrating effectiveness [32]. The cytotoxicity of antibody-based strategies towards cancer cells result from direct receptor blockade, induction of phagocytosis, and/or vascular cell ablation [32]. For these experiments, we used an anti-GPR171 antibody (ab60843; Abcam) raised against a peptide corresponding to residues 246–268 of the fourth extracellular domain of GPR171. Treatment with the anti-GPR171 antibody attenuated the proliferation of Calu-6 cells (Figure 3A), reducing cell viability to 28.2% (p = 7.2×10^−4^) of that in cells treated with control IgG after 4 days of treatment.

Next, we investigated the antitumor effects of targeting GPR171 *in vivo* using a Calu-6 xenograft model. BALB/c nude mice were subcutaneously injected with Calu-6 cells, then randomly distributed into control and anti-GPR171 antibody groups and treated by intravenous injection twice a week for 4 weeks. As expected, anti-GPR171 antibody treatment resulted in significant inhibition of Calu-6 xenograft tumors (p=0.026; Figure 3B and 3C).

The inhibition of cancer cell proliferation observed after knocking down GPR171 with shRNA or siRNA, or inhibiting it with a blocking antibody suggests that GPR171 is a promising anti-cancer target in lung cancer. To our knowledge, this is the first demonstration that GPR171 plays a tumor-promoting role by inducing cancer cell proliferation.

### GPR171 promotes invasion and migration of lung cancer cells

Metastasis of cancer cells is not a random process involving the simple spread of the initial tumor to a secondary site through the blood or lymphatic vessels [33]. For example, advanced lung cancer has a preference for spreading to bone, brain, liver, and adrenal gland [34]. Therefore, chemokine receptors that might serve a homing function have been the focus of efforts to elucidate the mechanism of organ-specific metastasis [33]. Notably, a number of GPCRs, including CXCR4, CXCR2 and EP receptors, have been implicated in the organ-specific metastasis of lung cancer [16, 19].

**Figure 4:**
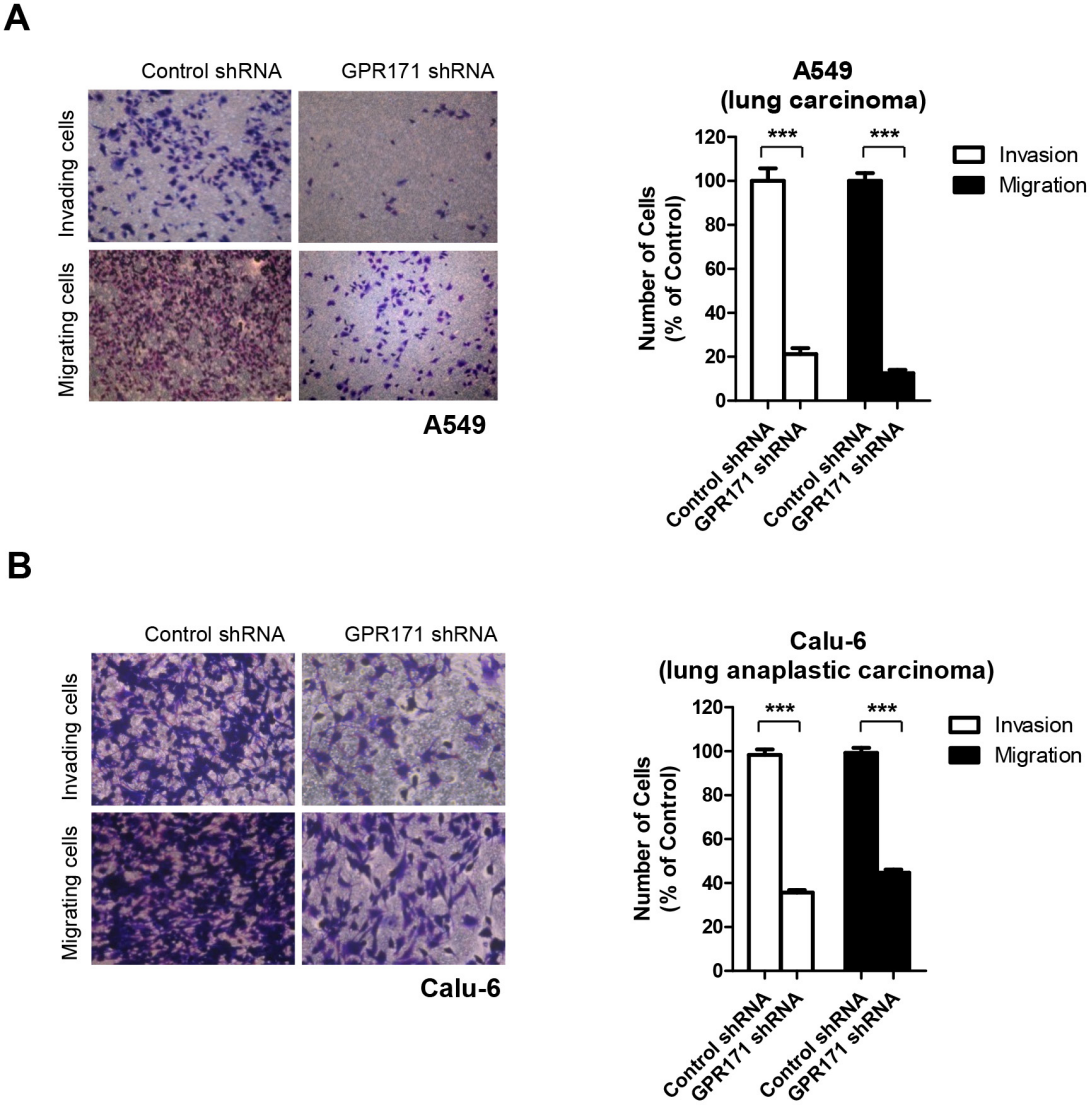
GPR171 triggers invasion and migration of lung cancer cells **A.** Invasion and migration assays of A549 cells expressing control or GPR171 shRNA. Top, invaded A549 cells expressing control or GPR171 shRNA were stained with crystal violet. Invasion was quantified by counting cells in five randomly selected regions. Bottom, migrated A549 cells expressing control or GPR171 shRNA were stained with crystal violet. Migration was quantified by counting cells in eight randomly selected regions. Results are presented as means ± SEM (error bars) (***P < 0.001, Student's t test). **B.** Invasion and migration assays of Calu-6 cells expressing control or GPR171 shRNA. Top, invaded Calu-6 cells expressing control or GPR171 shRNA were stained with crystal violet. Invasion was quantified by counting cells in five randomly selected regions. Bottom, migrated Calu-6 cells expressing control or GPR171 shRNA were stained with crystal violet. Migration was quantified by counting cells in five randomly selected regions. Results are presented as means ± SEM (error bars) (***P < 0.001, Student's t test).

We therefore examined the effect of GPR171 on metastasis, specifically cancer cell invasion and migration—a potential role of GPR171 that has not yet been examined. Indeed, we found that siRNA-mediated depletion of GPR171 in A549 cells caused approximately an 81% decrease in invasion (p = 2.0×10^−6^) and an 87% reduction in migration (p = 1.0×10^−7^) (Figure 4A). Similarly, we found that invasion and migration were reduced by 35.7% (p = 2.6×10^−6^) and 44.7% (p = 4.1×10^−6^), respectively, in GPR171-ablated Calu-6 cells compared with control cells (Figure 4B). These results suggest that GPR171 is involved in both metastasis and proliferation of lung cancer cells.

### Inhibition of GPR171 enhances the antitumor effects of an EGFR inhibitor

EGFR signaling is a major pathway of tumorigenesis in lung cancer. EGFR has the most frequent mutation rate (10-30%), compared with ALK (5%) and BRAF (2%) in NSCLC [35]. Our findings suggest that GPR171 could be another crucial tumorigenesis signaling pathway in lung cancer. Therefore, we sought to determine if GPR171 and EGFR signaling are interrelated in lung carcinogenesis. Notably, some GPCR ligands, prostaglandin E2 and bradykinin, activate MAPK via both EGFR-dependent and -independent mechanisms [7].

**Figure 5:**
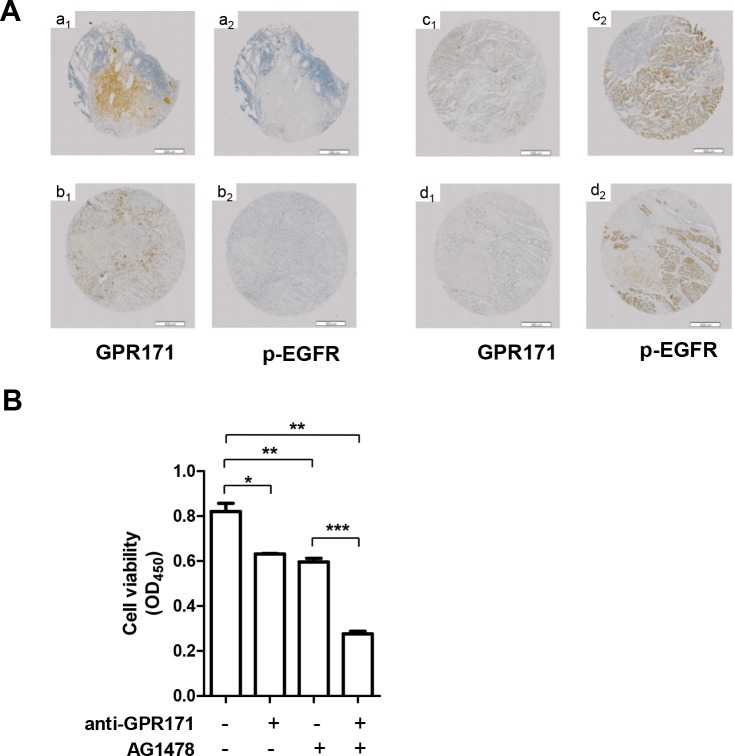
Inhibition of GPR171 enhances the anti-tumor effect of an EGFR inhibitor **A.** Immunohistochemical analysis of GPR171 and p-EGFR in lung cancer tissues.(a_1_, a_2_) small cell lung carcinoma;(b_1_, b_2_) adenocarcinoma of the lung; (c_1_, c_2_) bronchioloalveolar carcinoma; (d_1_, d_2_) adenocarcinoma of the lung. Scale bars = 500 μm. **B.** Cell viability assays of Calu-6 cells treated with 30 μg/ml of anti-GPR171 antibody in the presence or absence of 10 μM AG1478. Calu-6 cells were preincubated with AG1478 for 1 hour prior to incubation with anti-GPR171 antibody. Results are presented as means ± SEM (error bars) (n = 3; *P < 0.05, **P < 0.01 vs. untreated cells; ***P < 0.001 vs. AG1478 treated cells; Student's t test).

To determine if GPR171 exerts its tumor-promoting activity via an EGFR-dependent pathway, we compared GPR171 expression patterns with EGFR phosphorylation patterns (p-EGFR) in 47 lung cancer specimens using immunohistochemistry. In 22 of the 47 GPR171-expressing lung cancer tissues, no correlation was observed between GPR171 expression and p-EGFR. Specifically, high or moderate levels of GPR171 (Figure 5A, a_1_ and b_1_) were not correlated with EGFR activation (Figure 5A, a_2_ and b_2_), and high levels of p-EGFR (Figure 5A, c_2_ and d_2_) were not correlated with expression of GPR171 (Figure 5A, c_1_ and d_1_).

To test the efficacy of combined treatment with inhibitors of GPR171 and EGFR, we treated Calu-6 cells with an anti-GPR171 antibody in the presence or absence of AG1478, an EGFR-specific small molecule inhibitor. Treatment of cells with the anti-GPR171 antibody or AG1478 alone reduced cell viability by 23.0% (p = 0.019) and 27.3% (p = 9.3×10^−3^), respectively (Figure 5B). Combined treatment anti-GPR171 antibody and AG1478 led to a 66.3% reduction in cell viability (p = 1.5×10^−3^), indicating a synergistic effect.

Collectively, these findings indicate that combined inhibition of GPR171 and EGFR may be a promising strategy for lung cancer treatment, reflecting the fact that GPR171 induces tumorigenesis in an EGFR-independent manner. The possibility that other regulators, including ALK and BRAF, affect GPR171-induced tumorigenesis cannot be excluded.

## DISCUSSION

We identified a previously unrecognized role of GPR171 in lung cancer tumorigenesis. Specifically, we demonstrated that GPR171, also known as platelet-activating receptor H963, is a tumor-promoting gene that induces proliferation, invasion, and migration of lung cancer cells in an EGFR-independent manner. To date, inhibition of EGFR has been the most widely used targeted therapy in lung cancer. Our findings indicate that inhibition of GPR171 may be an important strategy for alternative or combinatorial therapy with EGFR inhibitors such as gefitinib, erlotinib, and cetuximab.

The only previously known function of GPR171 is its involvement in feeding and metabolism; thus, GPR171 has been suggested as a potential target for anti-obesity therapeutics [26]. Depletion of GPR171 causes an increase in food intake [26], providing a provocative link to the lung cancer-protective effect of being overweight suggested by meta-analyses [36]. In our experiments, the body weights of anti-GPR171 antibody-treated BALB/c nude mice were not significantly altered from those of control IgG-treated mice within a month (Supplementary Figure 3). Considering that 7-12 weeks are required for significant body weight changes after GPR171 shRNA virus infection [26], we cannot exclude the possibility of a link between GPR171 and body weight. Further studies are required to determine if GPR171 mediates the inverse relationship between obesity and tumorigenesis, because it is also widely accepted that obesity is a major risk factor for cancer that is associated with cancer development and recurrence, a worse prognosis, and overall mortality [37].

In cancers, specific neuropeptides result in autocrine and/or paracrine loops that promote sustained GPCR stimulation [22]. Since the peptide LENSSPQAPARRLLPP (BigLEN), a neuropeptide involved in feeding, activates GPR171, it would be interesting to test whether BigLEN is involved in GPR171-mediated tumorigenesis. Therefore, the presence of BigLEN might be a key to the tumorigenesis of lung cancer, and activation of GPR171, as well as the level of GPR171, might contribute to tumorigenesis. Further studies are needed to verify the mechanism by which GPR171 promotes proliferation and metastasis in lung cancer and to apply it for the development of a new anticancer drug.

## MATERIALS AND METHODS

### Cell culture

Calu-6 (ATCC HTB-56) and WI-38 (ATCC CCL-75) cells were maintained in MEMα containing 10% fetal bovine serum (FBS). A549 (ATCC CCL-185) cells were maintained in RPMI medium containing 10% FBS. IMR-90 (ATCC CCL-186) cells were maintained in Dulbecco's Modified Eagle Medium (DMEM) containing 10% FBS.

### Plasmids, transfection, and infection

pCMV6-GPR171 (sc115273) was purchased from OriGene. For GPR171 knockdown, five lentiviral pLKO.1 plasmids containing shRNAs against GPR171 sequences (TRCN0000009048, TRCN0000009049, TRCN0000009050, TRCN0000009051, and TRCN0000009052; Sigma) and two siRNAs against GPR171 sequences (1064445 and 1064442; Bioneer) were used. Cells were transfected using Nucleofector (Amaxa). Standard lentiviral techniques were used for shRNA infection. Cell viability was quantified using a Cell Counting kit-8 (CK04-11; Dojindo Molecular Technologies) with detection of absorbance at 450 nm using a microplate reader.

### Immunohistochemistry and immunoblotting

Four-micrometer-thick tissue sections were sliced onto silane coated micro slides and incubated at 60°C for 2 hour, deparaffinized by application of xylene (5 min x 3) and rehydrated. Endogenous peroxidase activity was quenched by incubating the sections in methanol with 0.3% H_2_O_2_ for 30 min. Antigen retrieval was performed by heating the slides in citrate buffer (0.01 M, pH 6.0) using a microwave in a pressure cooker for 15 min. After cooling for 2 hour at room temperature, immunohistochemical analysis was performed using anti-GPR171 antibody (ab60843, Abcam), anti-EGFR antibody (phosphor Y1068; Abcam) and UltraVision Quanto Detection System HRP DAB kit (Thermo Scientific), according to the manufacturer's instruction. Immunohistochemical analyses were blinded, and quantification carried out by two pathologists. The percentages of positive cells were scored and classified as follows: ≥50% as ‘strong’, 10-50% as ‘moderate’, and < 10% as ‘negative’. Tissue array slides (CCA4, lung cancer-metastasis-normal; SuperBioChips Laboratories) from multiple lung cancer patients were also used for immunostaining.

For immunoblotting, anti-GPR171 (GTX108131, GeneTex) and anti-tubulin (sc-5274) and anti-β-actin (sc-1616) from Santa Cruz Biotechnology were used.

### Xenograft studies

Calu-6 cells (1.0 × 10^6^) were suspended in phosphate-buffered saline (PBS) and injected subcutaneously into female BALB/c nude mice (n = 10). Mice were randomly distributed into control and antibody groups and administered normal rabbit IgG (2 mg/kg; Millipore 12-370) or anti-GPR171 antibody (2 mg/kg; Abcam ab60843) intravenously twice a week for the first 2 weeks. Tumor dimensions were measured with a caliper, and volumes were estimated using the formula, length (mm) × width (mm) × height (mm)/2.

### Invasion and migration assays

Cell invasion and migration assays were performed using a 24-well Transwell system (8 μm pore size; Costar 3422). For invasion assays, 2 × 10^5^ cells were plated in the upper chamber containing a Matrigel-coated membrane. For migration assays, 1 × 10^5^ cells were plated in the upper chamber containing a gelatin-coated membrane. In both assays, cells were plated in media without serum, and the lower chamber was filled with media supplemented with serum as a chemoattractant. After 48 hours, cells that had not invaded or migrated through the membrane pores were removed with a cotton swab. Cells in the membrane were stained with crystal violet (Sigma) and counted.

## SUPPLEMENTARY FIGURES AND TABLES


