# Supplementary material for: GPR171 expression enhances proliferation and metastasis of lung cancer cells
Source: Oncotarget. 2016 Jan 9;7(7):7856–65. doi: 10.18632/oncotarget.6856 (PMC4884959; doi:10.18632/oncotarget.6856)
Supplement: Supplementary file 1 [file oncotarget-07-7856-s001.pdf]

SUPPLEMENTARY FIGURES, TABLES

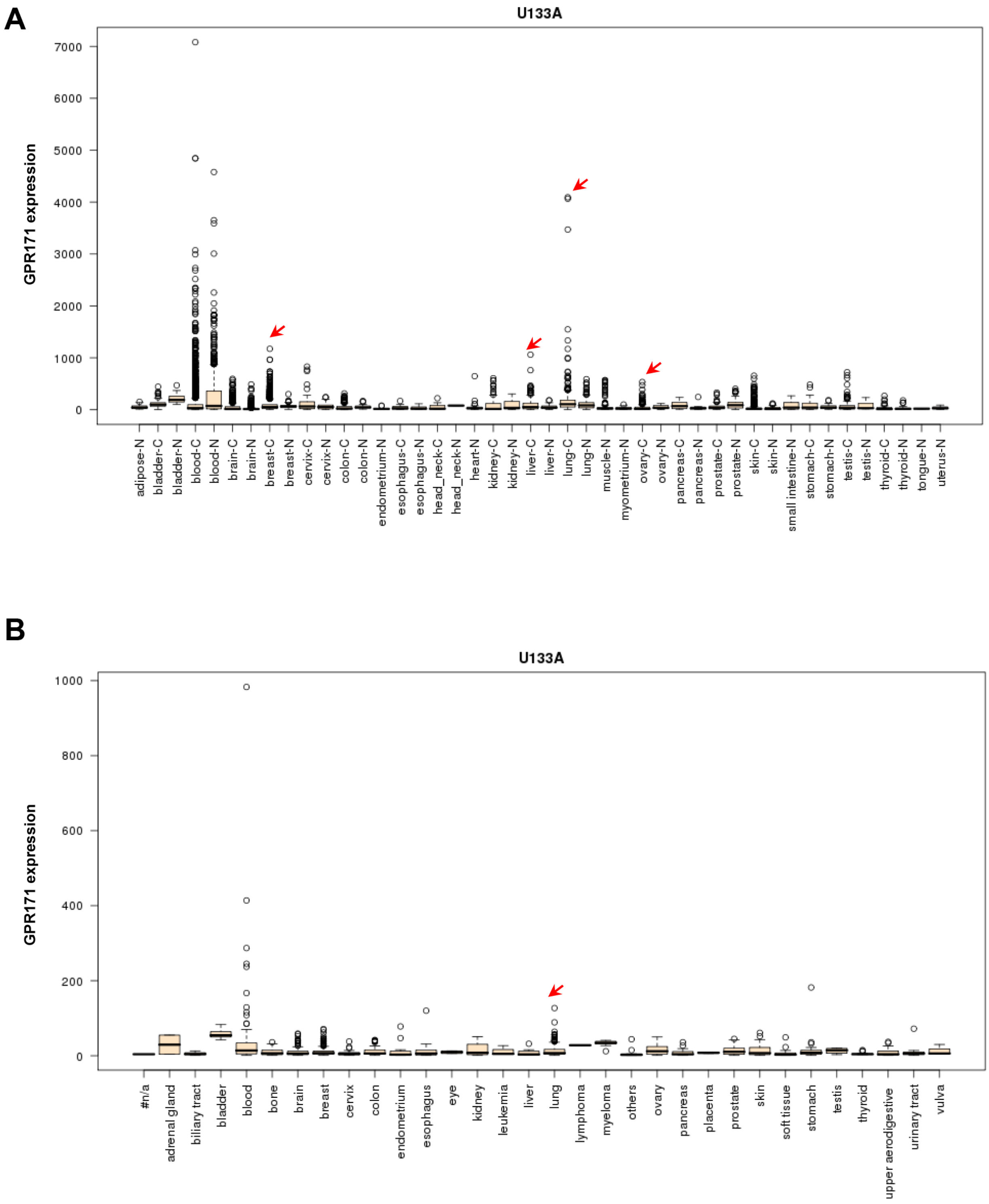

Supplementary Figure S1: Overexpression of GPR171 in a subset of cancers and cancer cell lines. A. Overexpression of GPR171 in breast, liver, lung, and ovarian cancers was assessed by web-accessible GENT microarray database (medical-genome.kribb.re.kr/GENT/). N indicates normal and C indicates cancer. Red arrows indicate overexpression in cancer tissues compared with normal tissues. **B.** Overexpression of GPR171 in a subset of cancer cell lines. Red arrow indicates overexpression in lung cancer cell lines.

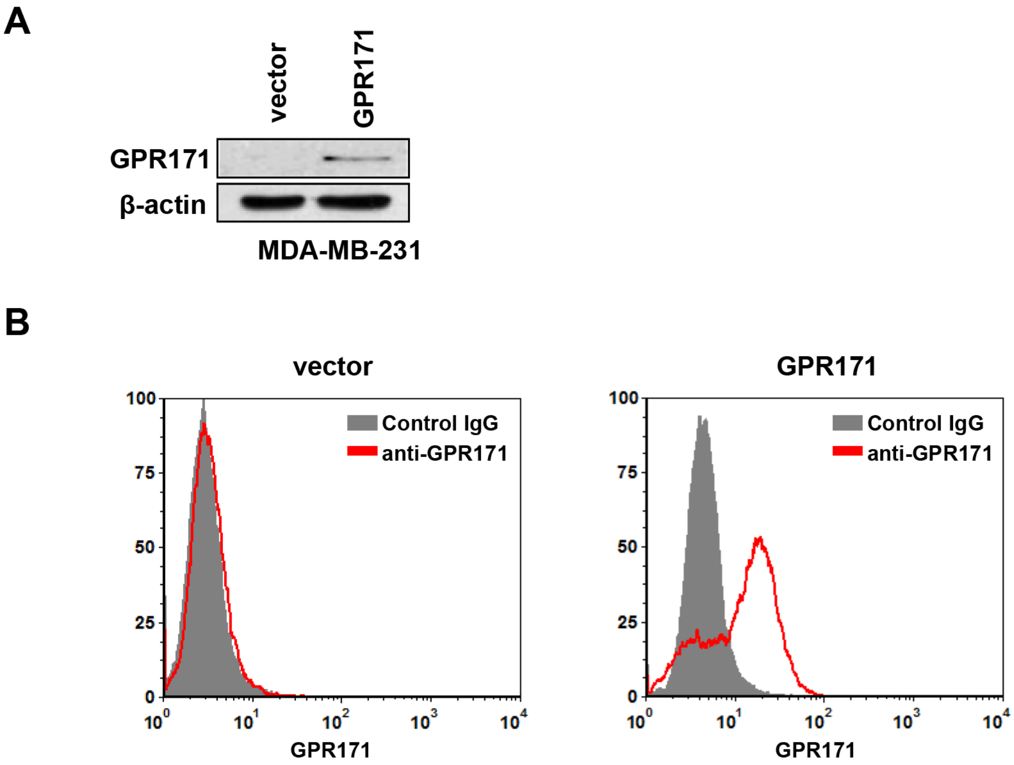

Supplementary Figure S2: The anti-GPR171 polyclonal antibody is specific to GPR171. A. Immunoblots of GPR171 and β-Actin from GPR171-negative MDA-MB-231 cells with or without ectopic expression of GPR171. Anti-GPR171 antibody (GTX108183; GeneTex) was used for the immunoblotting. **B.** Flow cytometry analysis of MDA-MB-231 cells expressing control vector or GPR171. Anti-GPR171 antibody (ab60843; Abcam) was used for the flow cytometry.

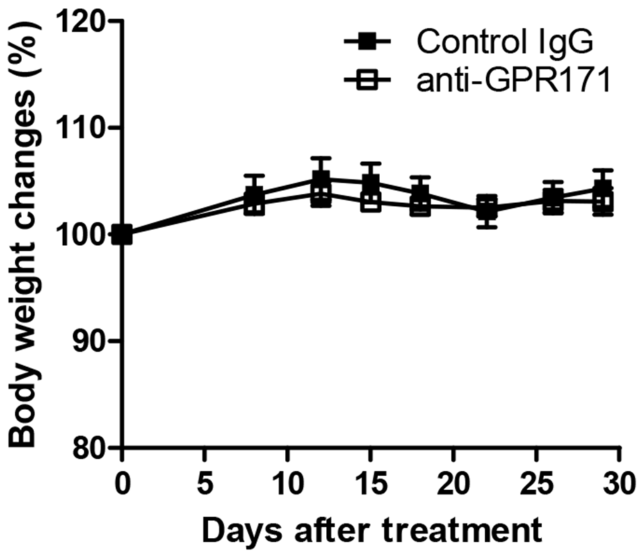

Supplementary Figure S3: No significant alteration of body weight in anti-GPR171 antibody-treated BALB/c nude mice compared to control IgG-treated mice within a month. Body weight changes (%) in mice with Calu-6 xenograft tumors were determined after treatment of anti-GPR171 antibody or control IgG. n = 5.

Supplementary Table S1: GPR171 is upregulated in NSCLC tissues

Clinical types of NSCLC	Number of stained tissues			Total positive tissues
	Strong	Moderate	Negative	
Squamous cell carcinoma	1/12	5/12	6/12	6/12
Adenocarcinoma	1/8	2/8	5/8	3/8
Bronchioloalveolar carcinoma	0/4	3/4	1/4	3/4
Adenosquamous carcinoma	1/3	1/3	1/3	2/3
Large cell carcinoma	1/3	1/3	1/3	2/3
Mucoepidermoid carcinoma	0/4	0/4	4/4	0/4
Adenoid cystic carcinoma	0/1	0/1	1/1	0/1

Immunohistochemical quantification of GPR171 in NSCLC tissues. Strong, moderate, and negative indicate >50%, 10-50%, and <10% positive cells, respectively.

Supplementary Table S2: GPR171 is upregulated in SCLC tissues

Clinical types of SCLC	Number of stained tissues			Total positive tissues
	Strong	Moderate	Negative	
Small cell carcinoma	1/2	1/2	0/2	2/2
Combined small cell and adenocarcinoma	0/1	0/1	1/1	0/1

Immunohistochemical quantification of GPR171 in SCLC tissues. Strong, moderate, and negative indicate >50%, 10-50%, and <10% positive cells, respectively.
